# Nanoscaled carborane ruthenium(II)-arene complex inducing lung cancer cells apoptosis

**DOI:** 10.1186/1477-3155-9-6

**Published:** 2011-02-22

**Authors:** Gen Zhang, Chunhui Wu, Hongde Ye, Hong Yan, Xuemei Wang

**Affiliations:** 1 State Key Lab of Bioelectronics (Chien-Shiung Wu Lab), Department of Biological Science and Medical Engineering Southeast University, Nanjing, 210096, PR China; 2 State Key Lab of Coordination Chemistry, School of Chemistry and Chemical Engineering, The Joint Laboratory of Metal Chemistry, Nanjing University, Nanjing, Jiangsu 210093, PR China

## Abstract

**Background:**

The new ruthenium(II)-arene complex, which bearing a carborane unit, ruthenium and ferrocenyl functional groups, has a novel versatile synthetic chemistry and unique properties of the respective material at the nanoscale level. The ruthenium(II)-arene complex shows significant cytotoxicity to cancer cells and tumor-inhibiting properties. However, ruthenium(II)-arene complex of mechanism of anticancer activity are scarcely explored. Therefore, it is necessary to explore ruthenium(II)-arene complex mechanism of anticancer activity for application in this area.

**Results:**

In this study, the ruthenium(II)-arene complex could significantly induce apoptosis in human lung cancer HCC827 cell line. At the concentration range of 5 μM-100 μM, ruthenium(II)-arene complex had obvious cell cytotoxicity effect on HCC827 cells with IC_50 _values ranging 19.6 ± 5.3 μM. Additionally, our observations demonstrate that the ruthenium(II)-arene complex can readily induce apoptosis in HCC827 cells, as evidenced by Annexin-V-FITC, nuclear fragmentation as well as DNA fragmentation. Treatment of HCC827 cells with the ruthenium(II)-arene complex resulted in dose-dependent cell apoptosis as indicated by high cleaved Caspase-8,9 ratio. Besides ruthenium(II)-arene complex caused a rapid induction of cleaved Caspase-3 activity and stimulated proteolytic cleavage of poly-(ADP-ribose) polymerase (PARP) *in vitro *and *in vivo*.

**Conclusion:**

In this study, the ruthenium(II)-arene complex could significantly induce apoptosis in human lung cancer HCC827 cell line. Treatment of HCC827 cells with the ruthenium(II)-arene complex resulted in dose-dependent cell apoptosis as indicated by high cleaved Caspase-8,9 ratio. Besides ruthenium(II)-arene complex caused a rapid induction of cleaved Caspase-3 activity and stimulated proteolytic cleavage of poly-(ADP-ribose) polymerase (PARP) *in vitro *and *in vivo*. Our results suggest that ruthenium(II)-arene complex could be a candidate for further evaluation as a chemotherapeutic agent for human cancers, especially lung cancer.

## Background

Enormous interest has been focused on the research of metallopharmaceuticals in order to find good alternatives to platinum drugs because of their significant clinical side effects and resistance that cause relapse of cisplatin[1]. In recent years, ruthenium complexes have attracted much interest because they exert their tumor-inhibiting effects by a mode of action different from that of Pt compounds[2]. Furthermore, they show a favorable toxicity profile in clinical trials: in the case of the ruthenium-indazole complex KP1019 only very moderate toxicities were observed in a dose range in which proteins were on average loaded with one ruthenium species, which should be sufficient for therapeutic activity[3].

Recently, potential bio-active moieties, such as carborane and ferrocene (Fc), have been extensively involved in new-type drug design because of their unique properties. Carboranes are carbon-containing polyhedral boron-cluster compounds with globular geometry. Novel carborane derivatives were synthesized to clarify its anti-cancer activity [4]. A myriad of compounds containing single- or multiple-carborane clusters were synthesized and evaluated in both cellular and animal studies[5]. Carboranes are a class of carbon-containing polyhedral boron-cluster compounds with remarkable thermal stability and exceptional hydrophobicity [6]. Carboranes have been tried to apply to the field of boron neutron capture therapy to incorporate large numbers of boron atoms into tumor cells[7]. Meanwhile, Fc has been incorporated in penicillin, chloroquine, tamoxifen, and diphenols thus modifying relative activities due to its small size, relative lipophilicity, ease of chemical modification, and accessible one-electron-oxidation potential[8,9]. Some unconjugated ferrocenyl derivatives and Fc-containing bioconjugates, have shown promising bioactivities like antineoplastic, antimalarial, or antibacterial activities.

**Figure 1:**
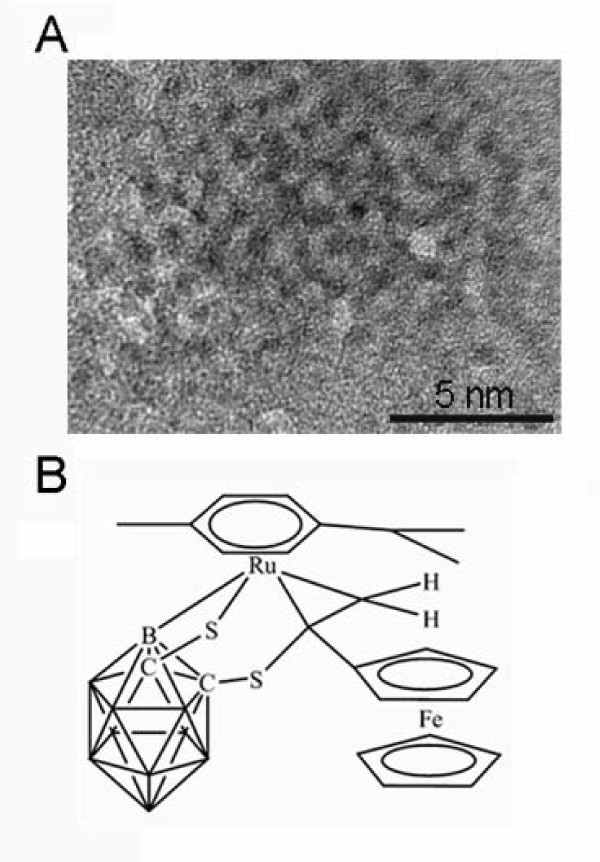
**Characterization of ruthenium (II)-arene complex**. (A) The transmission electron microscopy images of ruthenium (II)-arene complex. (B) The image structural of ruthenium (II)-arene complex.

Recent studies illustrate that a structural change from a Fc unit to a carboxyl group could lead to high selectivity toward cancer cells and facilitate the efficient inhibition of the proliferation of target cells, indicating that the tuning of the overall properties of the ruthenium(II)-arene complex by appropriate ligand tagging is critical to creating a selective anticancer agent[6]. In order to improve the activity of ruthenium (II)-arene complexes, which are of current interest as anticancer agents, the ruthenium (II)-arene complexes were synthesized by the reaction of ferrocenylacetylene in our work (Figure 1A). The ruthenium(II) arene fragment coordination with a multidrug resistance (MDR) modulator modified ligand (like anthracene) shows significant improvement of the cytotoxicity and P-glycoprotein inhibition behavior, demonstrating the promise of the ruthenium arene fragment in biomedical realm[10]. Research in progress is concerned with the development of advanced boron agents and neutron sources, other than nuclear reactors, for the treatment of a variety of cancer types using novel delivery methods[11].

However, ruthenium(II)-arene complex of mechanism of anticancer activity are scarcely explored and only a few dinuclear Ru complexes with tumor-inhibiting properties are known[12]. In this study, the new ruthenium(II)-arene complexes were observed to exhibit relatively high *in vitro *and *in vivo *sensitivity to HCC827 cells, resulting in dose-dependent cell apoptosis with a rapid induction of cleaved Caspase-3 activity and stimulated proteolytic cleavage of poly-(ADP-ribose) polymerase.

## Methods

### Cells, animals and chemicals

HCC827 (human lung cancer) cells purchased from the Institute of Hematology of Tianjin, Chinese Academy of Medical Sciences. The ruthenium (II)-arene complex (Figure 1B) was synthesized as our previous report[6]. Fetal calf serum was from Hyclone, RPMI 1640 cell culture medium Penicillin, streptomycin, 3-(4,5-Dimethyl-2-thiazolyl)-2,5-diphenyl-2H-tetrazolium bromide (MTT) (Gibco BRL, Grand Island, NY). Nude mice were provided by the Animal Feeding Farm of National Institute for the Control of Pharmaceutical and Biological Products (P.R. China). Annexin-V-FITC Apoptosis Detection Kit (Calbiochem, USA), Apoptotic DNA ladder Isolation Kit (BioVision, USA), antibody (Cell Signalling Technologies, USA) was purchased from Jinsite Biology Reagent Co.Ltd (Nanjing, China).

### Transmission Electron Microscopy

The ruthenium (II)-arene complex was observed under the transmission electron microscopy (Hitachi H-600-II) with an acceleration voltage of 200 kV.

### Cell growth inhibition study by MTT assay

Cells (2×10^3^/well) were plated in 100 μL medium/well in 96-well plates. After overnight incubation, MTT assays on HCC827 cells were treated with various concentrations of ruthenium (II)-arene complex. After treatment for 48 hour, 20 μL MTT solution (5 mg/ml) was added to each well. Four hours later, the supernatant was removed and 100 μL DMSO was added per well. Samples were then shaken for 15 min. Then the optical density (OD) was read at a wavelength of 540 nm. All experiments were performed in triplicate. Relative inhibition of cell growth was expressed as follows: % = (1-[OD]test/[OD]control)×100%.

### Flow cytometry analysis

Cells were seeded in 12 well plates at 1×10^5 ^cells per mL, 1 ml/well. After incubated for 72 h at 37°C, 5% CO_2_, HCC827 cells treated with relative ruthenium (II)-arene complex for 48 h.'Annexin-V-FITC Apoptosis Detection Kit' was used to determine apoptosis. Flow cytometric analysis was conducted using a FACSCalibur flow cytometer (BD Biosciences, USA).

### AO staining for apoptotic cells

Cancer cells were seeded in 6-well plates (5×10^5^/well) and incubated on relevant ruthenium (II)-arene complex for 72 h. To stain apoptotic cells, the cells were trypsinized and centrifuged for 6 min before 50 μL of AO dye mix (100 μg/mL acridine orange) was added to each well, and cells were viewed under fluorescence microscope.

### Intracellular ruthenium(II)-arene complex measurement

To measure intracellular ruthenium(II)-arene complex accumulation, HCC827 cells in 60-mm plates were incubated overnight in culture medium and then treated with ruthenium(II)-arene complex for 2 hours. Cells were harvested with a rubber scraper and centrifuged at 2,000 g for 10 min. The harvested cells were washed three times in cold PBS. The cells were digested to measure iron levels. Cells were dried at 105°C and ground in an agate mortar, and then digested in nitric acid. After appropriate dilution with doubly distilled H_2_O (ddH_2_O), the iron metal concentrations of the samples were determined by atomic absorption spectrophotometry using a TAS-986 spectrophotometer with respect to appropriate standard solutions in acidified ddH_2_O.

### Apoptotic DNA fragmentation analysis

The HCC827 cells were treated with various concentrations of the ruthenium (II)-arene complex for 72 h respectively. The cells without treated were considered as controls. Apoptotic DNA ladder of HCC827 cell was extracted using Apoptotic DNA ladder Isolation Kit, and then loaded onto 1% agarose gel. The DNA ladders stained with ethidium bromide were visualized under UV light.

### Apoptosis Western blotting analysis *in vitro*

HCC827 cells (1×10^5^/well) were plated in 2 mL medium/well in 6-well plates. After 72 hours treatment of relevant ruthenium (II)-arene complex at the concentration (100 μM, 50 μM) treatment, HCC827 cells lysates were prepared from treatment using modified RIPA lysis buffer. The lysates subjected to SDS-PAGE/Western blot analysis. The proteins were detected by enhanced chemiluminescence (ECL, GE Healthcare, NJ, USA). The following antibodies were used: anti-Cleaved Caspase-3, anti- Cleaved Caspase-9, anti- Cleaved Caspase-8, PARP, GAPDH levels were measured to ensure equal loading of protein.

### Experimental animals

HCC827 cells (4-5×10^6^) were suspended in 200 μL of culture medium and subcutaneously inoculated into the right flank of mice using a 1.0 mL syringe. Animals were kept in the facility with free access to food and water.

### Intravenous injection of reagents and tumor growth inhibition study

The nude mice inoculated with HCC827 cells were divided into 3 groups with seven mice in each group: (1) Control (n = 7); (2) 50 μmol/kg ruthenium (II)-arene complex (n = 7); (3) 100 μmol/kg ruthenium (II)-arene complex control (n = 7). When the tumor volume became around 50 mm^3 ^after one week of inoculation, treatment was injected for each group. Injection was intravenously administered by tail vein at day 0, 2, 4, 6, 8, 10, 12, 14, 16 and 18. The tumor volume of nude mice were measured and calculated at the 20th days after treatment. The tumor volume calculation was performed using the formula V = π/6×[(a+b)/2]^3^, where a is the largest and b is the smallest diameter of the tumor.

### Apoptosis Western blotting analysis *in vivo*

Briefly, the tumor tissues were removed from experimental mice. The tumors photographed, and then used for Western blot. The tumor lysis was subjected to Western blot analysis. And the proteins were detected by enhanced chemiluminescence (ECL, GE Healthcare, NJ, USA). The following antibodies were used: anti-Cleaved Caspase-3, anti- Cleaved Caspase-9, anti- Cleaved Caspase-8, PARP, GAPDH levels were measured to ensure equal loading of protein.

### In situ apoptosis by TUNEL staining

Apoptotic cell death in deparaffinized tumor tissue sections was detected using terminal deoxynucleotidyl transferase-mediated dUTP nick end-labeling (TUNEL) with the Klenow DNA fragmentation detection kit (Roche, USA). Briefly, sections were permeabilized with 20 μg/mL protease K, and endogenous peroxidase was inactivated by 3% H_2_O_2 _in methanol. Apoptosis was detected by labeling the 3'-OH ends of the fragmented DNA with biotin-dNTP using Klenow at 37ºC for 1.5 hours. The tumor slides were then incubated with streptavidin horseradish peroxidase conjugate, followed by incubation with 3,3'-diaminobenzidine and H_2_O_2_. Apoptotic cells were identified by the dark brown nuclei observed under light microscope.

### Statistical analysis

Results were presented as Mean ± SD. A t-test was performed in each group for each time point. A value of *P *< 0.05 was considered statistically significant.

## Results

### **Cytotoxicity of ruthenium(II)-arene complex on HCC827 cells**

**Figure 2:**
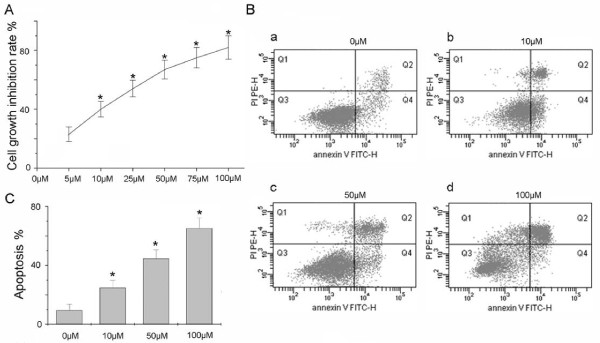
**Measurement of cell apoptosis rate**. (A) HCC827 cells MTT assay (1) 5 μM; (2) 10 μM; (3) 25 μM; (4) 50 μM; (5) 75 μM; (6) 100 μM. The ruthenium (II)-arene complex treatment time was 48 hours. * *P *< 0.05, compared to the (1) treatment. (B) HCC827 cells detected by Flow Cytometry using Annexin-V-FITC method. (a) control treatment; (b) 10 μM; (c) 50 μM; (d) 100 μM. The ruthenium (II)-arene complex treatment time was 36 hours. (C) Quantitative analysis of apoptotic cells after various treatments shown in B. * *P *< 0.05, compared to the control treatment.

Initially, the synthesized ruthenium (II)-arene complex was characterized by transmission electron microscopy. The average size of the ruthenium (II)-arene complex was about 1 nm (Figure 1A). The MTT assay was carried out for the cells cytotoxicity study. The cells were treated with different concentrations of ruthenium (II)-arene complex (10 μM-100 μM) for 48 h. As shown in Figure 2A, HCC827 cells viability was significantly reduced after 48 h exposure to ruthenium (II)-arene complex. Those cells growth inhibition were increased in a dose-dependent manner. The IC _50 _values of each treatment were calculated, as shown in (Table 1).

**Table 1:** IC_50 _values of ruthenium (II)-arene complex according to the MTT assays

	**IC**_**50 **_**values**
	
Treatment type	HCC827 cells
Ruthenium (II)-arene complex	19.6 ± 5.3 μM

The values are shown as mean ± SD, *n *= 5.

Note: IC_50 _values indicate the 50% growth inhibitory concentration values of ruthenium (II)-arene complex.

### Cell apoptosis rate induced by Ruthenium (II) Arene Complex

**Figure 3:**
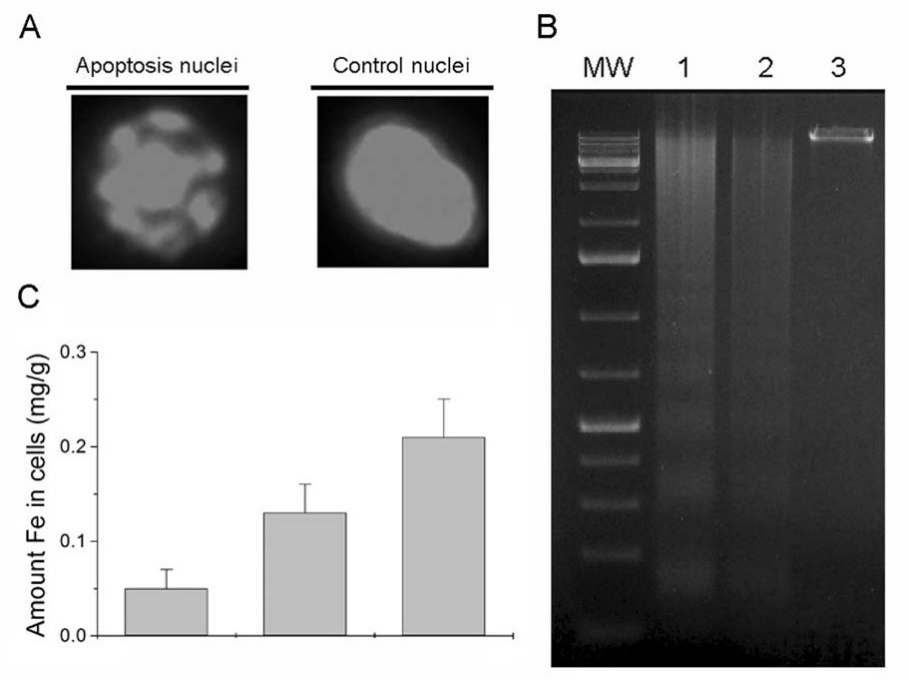
**Morphological images and genomic DNA apoptosis and Cyclic voltammetry study**. (A) Detection of apoptotic and normal cells by Acridine Orange Staining. Apoptotic nuclei could be identified by their distinctively marginated and fragmented appearance. (B) The genomic DNA was isolated from the HCC827 cells that underwent various treatments. The DNA ladders were visualized under UV light. Lane M: Molecular weight markers; Lane 1: Cells treated with 100 μM ruthenium (II)-arene complex; Lane 2: Cells treated with 50 μM ruthenium (II)-arene complex; Lane 3: DNA isolated from HCC827 cells without any treatment. (C) Cyclic voltammetry study of ruthenium (II)-arene complex residue outside HCC827 cells after incubating. (a) ruthenium (II)-arene complex (10 μM); (b) ruthenium (II)-arene complex (10 μM) and cells for 1 h; (c) ruthenium (II)-arene complex (10 μM) and cells for 2 h. Pulse amplitude: 0.05 V; pulse width: 0.05 s; pulse period: 0.2 s.

Figure 2B shows that relevant ruthenium (II)-arene complex induced a much higher cell apoptosis rate than untreated control using Annexin-V-FITC apoptosis detection method. We found that the percentage of apoptotic cells was 8.9%, 25.2%, 43.4%, 65.2% for the treatment with 0 μM, 10 μM, 50 μM, 100 μM ruthenium (II)-arene complex, respectively (Figure 2C). Ruthenium (II)-arene complex demonstrated a sustained, dose-dependent anti-proliferative activity in HCC827 cells. Using acridine orange staining for apoptotic cells, apoptotic nuclei were identified by their distinctively marginated and fragmented appearance under fluorescence microscope. The apoptotic nuclei of HCC827 cells (Figure 3A, Apoptosis nuclei) at 72 hours could be identified by their distinctively marginated and fragmented appearance. For the control cells without treatment, cells nuclei were normal as shown in (Figure 3A, control nuclei). In summary, each of the experimental methods performed demonstrated a substantial increase in cell apoptosis following treatment of HCC827 cells with relevant ruthenium(II)-arene complex.

### Apoptotic DNA fragmentation

To determine whether the cell cytotoxicity was due to the apoptotic response, the DNA fragmentations were examined by agarose gel electrophoresis. When HCC827 cells were treated with 100 μM ruthenium (II)-arene complex, the intensity of fragmented chromosomal DNA bands was much higher than that observed from cells treated with 50 μM ruthenium (II)-arene complex (Figure 3B, lane 1, 2 respectively). These results provide the evidence that the remarkable enhancement of apoptosis was induced by the anticancer effect of relevant ruthenium (II)-arene complex on HCC827 cells.

### Intracellular ruthenium(II)-arene complex measurement

HCC827 cells uptake of ruthenium(II)-arene complex was examined after 2 hours treatment. Figure 3C shows the Fe levels in various treatments. When ruthenium(II)-arene complex were injected into cells, the amount of Fe in tested cells was significantly higher than those in the control group. In accordance with this results, we demonstrate that ruthenium (II)-arene complex was intaken by cellular behavior with concentration-dependent used in the experiment.

### Activation of the signal pathway by ruthenium(II)-arene complex in HCC827 Cells

**Figure 4:**
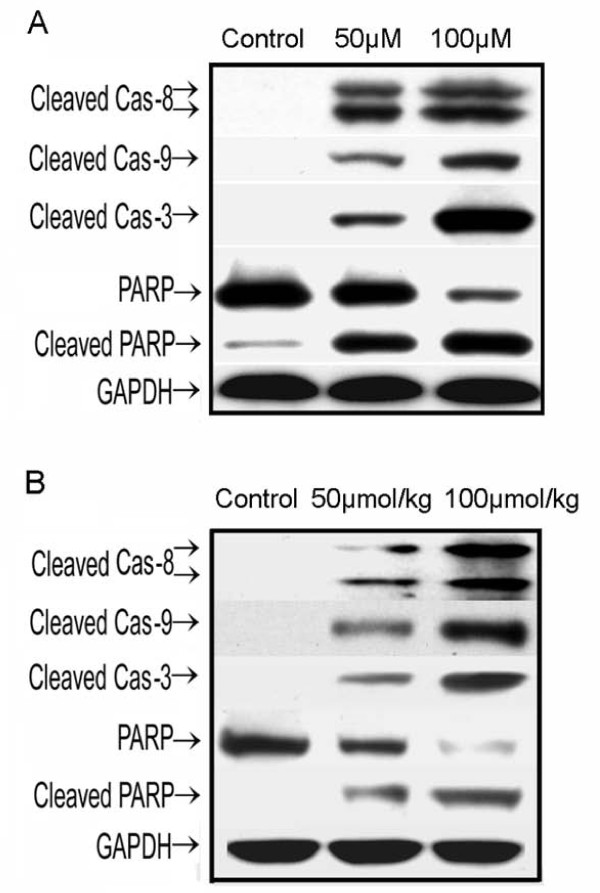
**Activation of the apoptosis signal pathway**. (A) Western blotting *in vitro*. Cell lysates were prepared from the cells treated with 50 μM ruthenium (II)-arene complex, 100 μM ruthenium (II)-arene complex. HCC827 cells without treatment were used as a control. The following antibodies were used: anti-cleaved Caspase-8, anti-cleaved Caspase-9, anti-cleaved Caspase-3, and anti-PARP antibody. GAPDH was served as a loading control. (B) Western blotting *in vivo*. Tumor lysates were prepared from the cells treated with 50 μmol/kg ruthenium (II)-arene complex, 100 μmol/kg ruthenium (II)-arene complex. HCC827 cells without treatment were used as a control. The following antibodies were used: anti-cleaved Caspase-8, anti-cleaved Caspase-9, anti-cleaved Caspase-3, and anti-PARP antibody. GAPDH was served as a loading control.

To further understand the molecular mechanisms underlying the ruthenium(II)-arene complex mediated apoptosis in HCC827 cells, we investigated apoptosis-related protein expression in HCC827 cells. When HCC827 cells were treated with 100 μM ruthenium (II)-arene complex, the cleaved Caspase-8 (p43/p41) signals on Western blots were much stronger than those for cells treated with 50 μM ruthenium(II)-arene complex (Figure 4A). Similar results were obtained for cleaved Caspase-9 and Caspase-3. PARP is a known downstream target of active Caspase-3 and can be cleaved during the induction of apoptosis. In HCC827 cells treated with 100 μM ruthenium (II)-arene complex, the cleavage of PARP via the proteolytic degradation of most length PARP into the activated form was detected along with Caspase-3 activation. Treatment of cells with 50 μM ruthenium (II)-arene complex initiate length PARP into the activated form was detected along with Caspase-3 activation. These data suggest that ruthenium (II) arene complex treatment induces cell apoptosis by increasing activation of Caspase-8, 9 pathways in HCC827 cells.

### Analysis of cell apoptosis in HCC827 xenograft tumors

**Figure 5:**
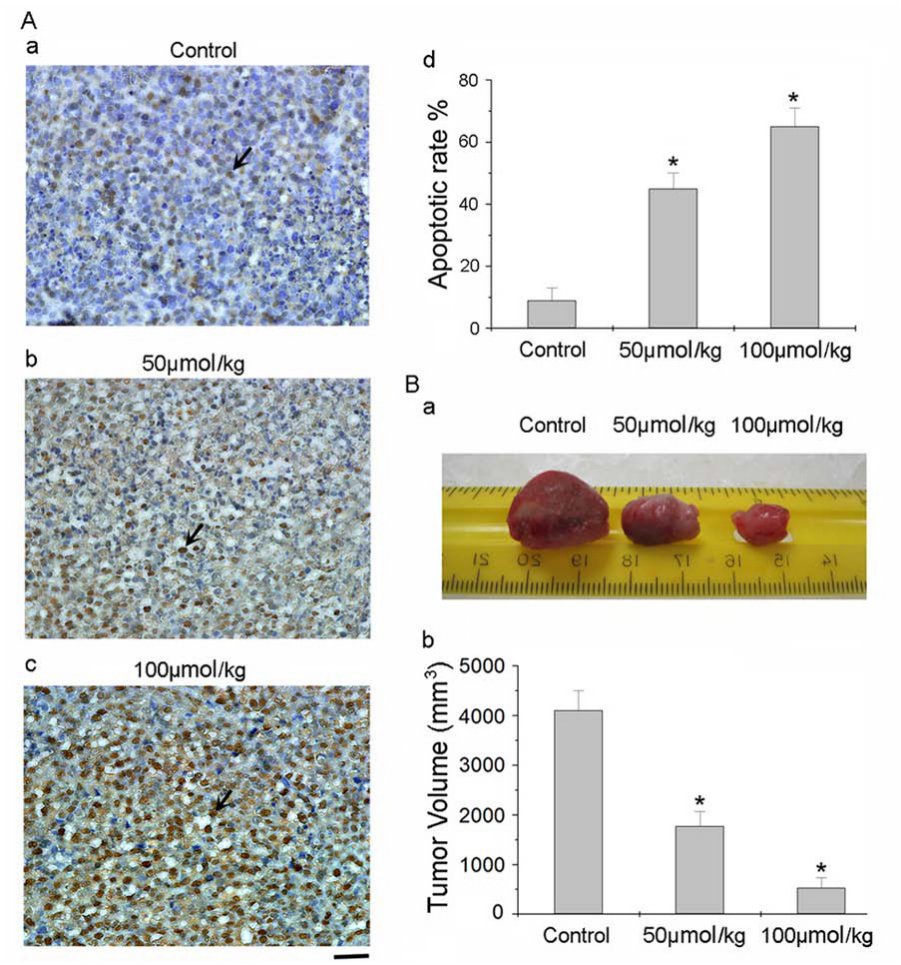
**Immunohistochemical staining of apoptotic cells in HCC827 xenograft tumors**. (A) TUNEL staining was performed on tissue sections of HCC827 xenograft tumors treated as follows: (a) group 1, control group; (b) group 2, 50 μmol/kg ruthenium (II)-arene complex treatment; (c) group 3, 100 μmol/kg ruthenium (II)-arene complex treatment. Arrows: TUNEL positive cells (indicate apoptotic cells). Scale bars: 50 μm. (d) Quantitative analysis of apoptotic cells after various treatments shown in (Figure 5. A a, b, c). (B) Inhibition of tumor growth in HCC827 nude mice with different treatments. (a) The different treatment effects on the tumor growth inhibition in nude mice inoculated with HCC827 cells: group 1, no treatment, serve as a control group; group 2, 50 μmol/kg ruthenium (II)-arene complex treatment; group 3, 100 μmol/kg ruthenium (II)-arene complex. (b) Quantitative analysis of tumor volume after various treatments shown in (Figure 5. B a). Each data represents the mean ± standard deviation (n = 7). *Indicates significant difference in comparison to the control group (p < 0.05).

The anticancer effect of ruthenium (II)-arene complex on the apoptosis induction in the xenograft tumors excised from HCC827 nude mice was evaluated. The apoptotic rate in the HCC827 xenograft tumor on the control group (Figure 5A, a) was about 9.1%. The apoptotic rate in group 2 (HCC827 mice treated with 50 μmol/kg ruthenium (II)-arene complex) did increase to about 46.7% significantly (Figure 5A, b). For group 3 (HCC827 mice treated with 100 μmol/kg ruthenium (II)-arene complex), as compared to that of the control group, the number of apoptotic cells were greatly increased to about 65.9% (Figure 5A, c).

It is clear from Figure 5B that the tumor volume in HCC827 nude mice the control group were the largest (4000 mm^3^, group 1). 50 μmol/kg ruthenium (II)-arene complex greatly inhibited the tumor growth in HCC827 nude mice (1800 mm^3^, group 2). In contrast to group 2, 100 μmol/kg ruthenium (II)-arene complex greatly showed much more tumor growth inhibition in HCC827 nude mice (500 mm^3^, group 3). Those results provide the fresh evidence that remarkable enhancement of cell apoptosis can be readily induced and tumor growth inhibition can be inhibited by anticancer effect of ruthenium (II)-arene complex *in vivo*.

### Antitumor signal pathway of ruthenium (II)-arene complex *in vivo*

To further investigate whether the antitumor signal pathway of ruthenium (II)-arene complex investigated in a lung tumor model using HCC827 nude mice is consistent with signaling apoptosis induction *in vitro*, tumor samples from xenograft mice were subjected to Western blot assays. Western blots using the tumor extracts showed that 50 μmol/kg ruthenium (II)-arene complex treatment increased relatively subsequent half of PARP activation, whereas 100 μmol/kg Ruthenium (II) Arene Complex treatment led to most activation of PARP when compared with control (Figure 4B). PARP is a known downstream target of cleaved Caspase-3. Caspase-3 is a known downstream target of active Caspase-8, 9. Similar results were obtained for cleaved Caspase-8, 9, 3. When HCC827 cells were treated with 100 μmol/kg ruthenium (II)-arene Complex, the cleaved signals on western blots were much stronger than those for cells treated with 50 μmol/kg Ruthenium (II) Arene Complex.

## Discussion

The new ruthenium (II)-arene complex was about 1 nm, ruthenium (II)-arene complex has nanocomposites and biomaterials activity. So the complex may be used as nanodrug, for cancer targeting, and intracellular labeling. Although ruthenium (II)-arene complex is used as an anti SMMC-7721 and HELF cancer drug[6], our group, along with several others, reported that new ruthenium (II)-arene complex could be used as an anti lung cancer drug. Ruthenium (II)-arene complex could induce cancer cells apoptosis, the underlying molecular mechanism to HCC827 cells was unclear. One possible mechanism is that enhanced biomolecular recognition and transportation through the cell membrane because of the hydrophobic face provided by the arene ligands[13]. Consistent with previous observations, our current study indicates that ruthenium (II)-arene complex treatment activated Caspase-8, 9 pathways to induce apoptosis in HCC827 cells.

Apoptosis is an important biological process in many systems and can be triggered by a variety of stimuli received by the cells[14]. Caspase-family represents the key components of the apoptotic machinery within the cells and consists of at least 14 different caspase proteases in mammals [15]. There are two major pathways of caspase activation with one of which is initiated by TNF-family receptors that recruit several intracellular proteins to form a ''death-inducing signaling complex'' (DISC) upon external "death signal" stimulation, leading to Caspase-8 activation and apoptosis; another major apoptosis pathway targets mitochondria that release Cytochrome c activates pro-Caspase-9; the initiator Caspases, such as Caspase 8 and 9, activated via these two pathways, can cleave and activate their downstream effector Caspases, such as Caspase-3, thus propagating a cascade of proteolysis that results in apoptosis [16].

Firstly, we analyzed the cells apoptosis morphology from various angles regarding MTT assay, nuclei staining, DNA fragment assay. We demonstrated in this study that ruthenium (II)-arene complex elicited an anti-proliferative effect in dose-dependent manner in HCC827 human lung cancer cells. The apparent IC_50 _values for relevant ruthenium (II)-arene complex are estimated as 19.6 μM for HCC827 cells. In addition, when cells were treated with ruthenium (II)-arene complex, they exhibited characteristic morphological features of apoptosis, such as chromosomal condensation and DNA fragment. With flow cytometry assay, we analyze quantitative apoptotic cells after various treatments. So the new ruthenium (II)-arene complex could be used as inducing HCC827 cells apoptosis with relatively low concentration. We also demonstrate that ruthenium (II)-arene complex was intaken by cellular behavior with concentration-dependent used the spectrophotometry experiment. So ruthenium (II)-arene complex is effective chemical for anticancer due to the unique properties of the respective material at the nanoscale level.

In the current study, we found that ruthenium (II)-arene complex treatments activated Caspase-8, 9 pathways to induce apoptosis in HCC827 cells. Cleaved Caspase-8, Caspase-9 activated Caspase-3 that correlated with the increased cleaved PARP expression after ruthenium (II)-arene complex treatments (Figure 4A). And then DNA fragmentation is induced during the cells apoptosis by cleaved PARP expression. To further investigate whether the antitumor signal pathway of ruthenium (II)-arene complex investigated in a lung tumor model using HCC827 nude mice are consistent with signaling apoptosis induction *in vitro*, tumor samples from xenograft mice were subjected to Western blot assays (Figure 4). The results provide the evidence that the same antitumor signal pathway of ruthenium(II)-arene complex induced in the HCC827 cells *in vitro *and *in vivo*. Consequently, as shown in Figure 5, TUNEL results demonstrate that tumor growth inhibition is inhibited by apoptosis effect of ruthenium (II)-arene complex *in vivo*. Therefore, the ruthenium (II)-arene complex treatment might lead to the upregulation of some TNF-family receptors activity and Cytochrome c binds to the Caspase activator, which in turn may lead to the degradation of the Caspase-8, 9 respectively. The *in vitro *and *in vivo *assay results of the current study further support this degradation of the Caspase protein, suggesting the crucial role of the ruthenium (II)-arene complex to induce cancer cell apoptosis.

## Conclusion

In summary, our results show that the nanoscale level of ruthenium (II)-arene complex induced significant apoptosis, and activation of Caspases in HCC827 cancer *in vitro *and *in vivo*. The underlying apoptotic mechanism was revealed to be crucially dependent on the activation of Caspase-8, 9 that engaged at later stage at least. These observations suggest that ruthenium (II)-arene complex is a potential candidate for lung cancer chemotherapy. Those results provide the evidence that remarkable enhancement of apoptosis can be induced and tumor growth can be inhibited by anticancer effect of ruthenium (II)-arene complex *in vivo*. It is evident that the better understanding of the apoptotic mechanism and possible chemotherapeutic activity of ruthenium (II)-arene complexs chemotherapeutic activity would benefit the future clinical study.

## Abbreviations

MTT: 3-(4,5-dimethylthiazol-2-yl)-2,5-diphenyl tetrazolium bromide; PARP: proteolytic cleavage of poly-(ADP-ribose) polymerase; TUNEL: terminal deoxynucleotidyl transferase-mediated dUTP nick end-labeling;

## Competing interests

The authors declare that they have no competing interests.

## Authors' contributions

HY, HDY synthesized ruthenium (II)-arene complex; GZ, CHW performed the cell and the animal studies; GZ, XMW wrote the manuscript. All authors read and approved the final manuscript.
